# Clinical Characteristics and Outcomes of Pediatric Cerebral Venous Sinus Thrombosis: An Analysis of 30 Cases in China

**DOI:** 10.3389/fped.2019.00364

**Published:** 2019-09-04

**Authors:** Xin-Hua Wang, Lin-Mei Zhang, Yi-Ming Chai, Ji Wang, Li-Fei Yu, Shui-Zhen Zhou

**Affiliations:** Department of Neurology, Children's Hospital of Fudan University, Shanghai, China

**Keywords:** stroke, cerebral venous sinus thrombosis, CVST, child, retrospective studies

## Abstract

**Background:** Cerebral venous sinus thrombosis (CVST) in children is rare in a clinical setting. The aim of this study was to summarize the etiological, clinical, and imaging characteristics of CVST in children.

**Methods:** We retrospectively analyzed the data of 30 patients with a diagnosis of CVST who were admitted to Children's Hospital of Fudan University from 2008 to 2018. The medical records, including clinical manifestations, laboratory data, neurological findings, treatment, and short-term prognosis were analyzed.

**Results:** Etiologically, the causes of CVST were infection (7/30), tumor (3/30), nephritis or nephrotic syndrome (8/30), traumatic brain injury (1/30), and undefined disease (11/30). All 30 cases were diagnosed with CVST after a neuroimaging examination using brain magnetic resonance imaging (MRI) combined with magnetic angiography venography (MRV). With regard to short-term prognosis, all the patients were treated with anticoagulants, after which 26 cases improved.

**Conclusions:** CVST patients do not typically present with specific clinical manifestations, which leads to a high rate of misdiagnosis and delayed therapy. Increased consideration and prompt MRV checkup plays a key role in achieving an accurate diagnosis. Overall, anticoagulation is a safe and effective treatment for CVST.

## Introduction

Cerebral venous sinus thrombosis (CVST) is a particular type of cerebrovascular disease accompanied by focal cerebral edema. Intracranial hypertension, venous cerebral infarction, and seizures are the most prominent clinical features of CVST (1, 2). Neonates, in general, have a greater risk of poor outcomes, while children show a better outcome and make a full recovery (3). A Canadian study showed that the incidence of CVST in children under the age of 18 years was 0.67/100,000 (4). Due to the large population of China, CVST is a treatment challenge for Chinese clinicians. In recent years, with the widespread application of magnetic resonance imaging (MRI), especially magnetic resonance venography (MRV) technology, the diagnostic rate of this disease has continuously improved (5). At present, there are few reports of this disease from specialized children's hospitals. To further our understanding of this disease and to help reduce the rate of misdiagnosis, we retrospectively analyzed the clinical data of pediatric cases of CVST in patients admitted to our hospital within the past 10 years.

## Materials and Methods

### Subject Enrollment and Data Extraction

Medical records of patients admitted to our hospital between 2008 and 2018 were retrospectively reviewed to identify potential subjects. All patients <18 years old with CVST confirmed by MRV were included in this study. No exclusion criteria were used. We collected the following types of data from the medical records: (i) general conditions, including gender, age, and past history; (ii) disease state including the time from onset to diagnosis, etiology, clinical symptoms, and signs; (iii) imaging features; (iv) laboratory tests, including platelets, white blood cells, C-reactive protein, and coagulation function; (v) short-term prognosis (state at discharge).

The study was approved by the ethics committee at Children's Hospital of Fudan University. Due to the retrospective design of this study, informed consent was waived.

### Statistical Analysis

Continuous data were expressed as median and interquartile range and categorical data were expressed as absolute numbers and percentages. Statistical Package for the Social Sciences software (version 25.0) was used for data analysis.

## Results

### Clinical Data

Thirty subjects were identified based on the selection criteria. Table 1 lists the summary of all patients. There were 16 males and 14 females, with a median age of 8.33 years. The median duration of CVST was 14 days and only 10 patients were diagnosed within 7 days of onset. The clinical manifestations included headache (89%); vomiting (73%); weakness (17%); tic (30%); coma (23%); fever (13%); dyskinesia (14%); and visual symptoms (41%) including blurred vision, blindness, double vision, swollen eyelids, and eye pain accompanied by protrusion.

**Table 1:** Characteristics of the subjects.

	***N***	**Results**
Age (years)	30	8.33 (3.44–12.67)
Male (%)	30	16 (53%)
Female (%)	30	14 (47%)
Duration (days)	30	14 (2–1095)
Signs and symptoms		
Headache (%)	28	25 (89%)
Vomiting (%)	30	22 (73%)
Visual symptoms (%)	29	12 (41%)
Weak (%)	29	5 (17%)
Seizures (%)	30	9 (30%)
Coma (%)	30	7 (23%)
Fever (%)	30	4 (13%)
Dyskinesia (%)	29	4 (14%)
Etiologies		
Infection (%)	30	7 (23%)
Tumor (%)	30	3 (10%)
Nephritis or nephrotic syndrome (%)	30	8 (27%)
Traumatic brain injury (%)	30	1 (3%)
Unknown (%)	30	11 (37%)
Lesion site on MRV		
Superior sagittal sinus (%)	20	15 (75%)
Inferior sagittal sinus (%)	20	2 (10%)
Straight sinus (%)	20	6 (30%)
Left transverse sinus (%)	20	12 (60%)
Right transverse sinus (%)	20	14 (70%)
Left sigmoid sinus (%)	20	7 (35%)
Right sigmoid sinus (%)	20	9 (45%)
Cavernous sinus (%)	20	1 (5%)
Parenchymal involvement (%)	20	1 (5%)
Laboratory tests		
D-dimer (mg/L)	20	1.65 (0.61–2.97)
Prothrombin time (seconds)	20	12.8 (11.4–13.7)
Active partial thrombin time (seconds)	20	31.2 (26.9–36.0)
Thrombin time (seconds)	20	17.6 (15.9–19.7)
Fibrinogen (g/L)	20	3.41 (2.81–4.61)
Hemoglobin (g/L)	19	128 (115–140)
White blood cell (10^9^/L)	19	7.9 (5.4–12.9)
Platelet (10^9^/L)	19	263 (192–386)
C-reactive protein (mg/L)	19	8 (8–8)
Outcome		
Died (%)	30	2 (7%)
Alive (%)	30	26 (87%)
Transferred (%)	30	2 (7%)

*Continuous data were expressed as median and interquartile range and categorical data were expressed as absolute number (percentage)*.

### Imaging

**Figure 1:**
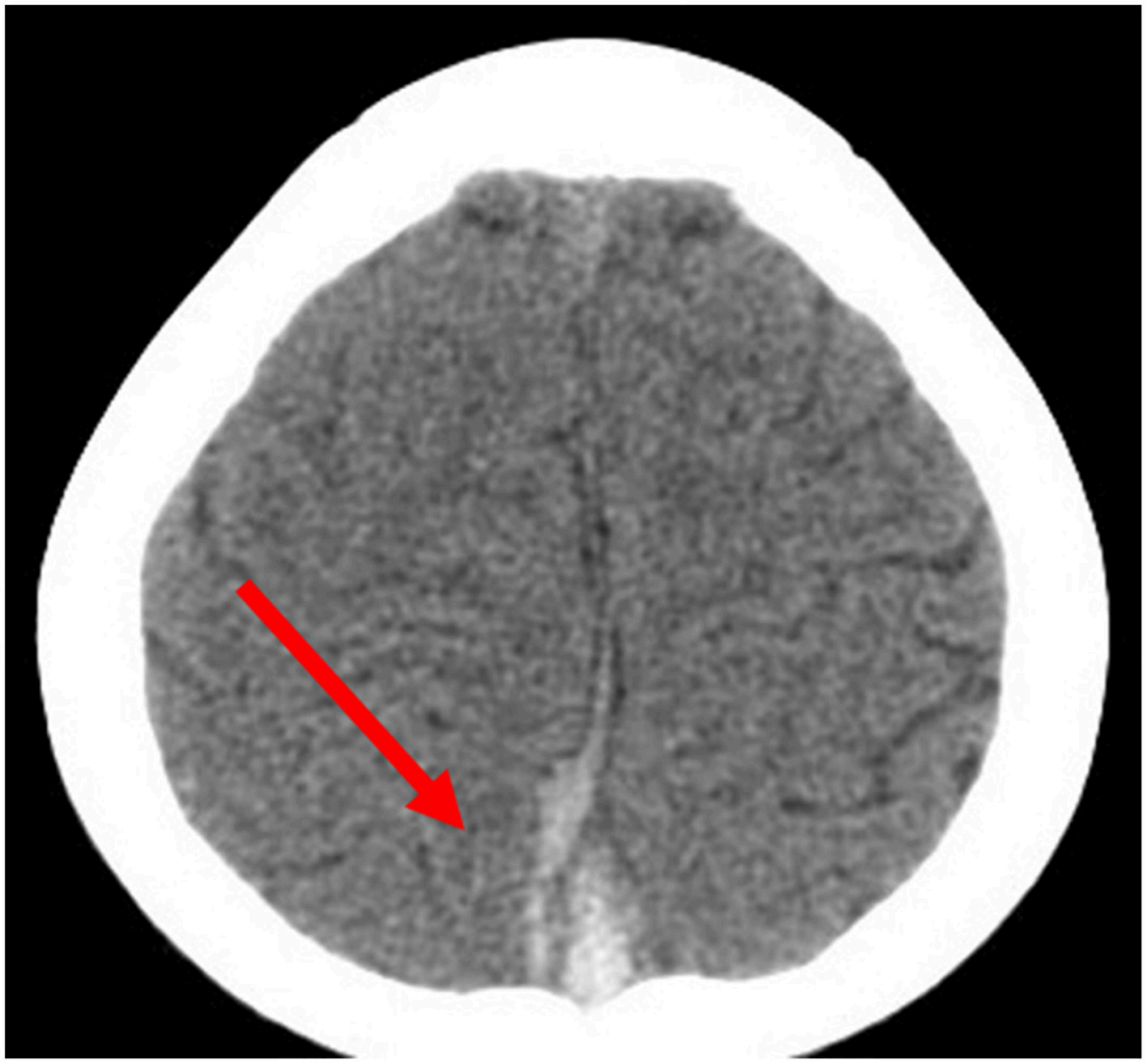
A CT scan before treatment showing a high-density shadow in the superior sagittal sinus.

**Figure 2:**
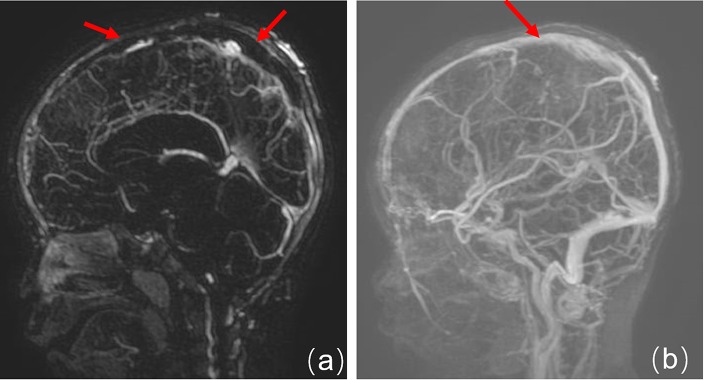
Enhanced MRV. **(a)** Before treatment, the superior sagittal sinus is not filled and shows a long strip filling defect. **(b)** At 5 months after treatment, the imaging shows the upper sagittal sinus development and local irregularities.

**Figure 3:**
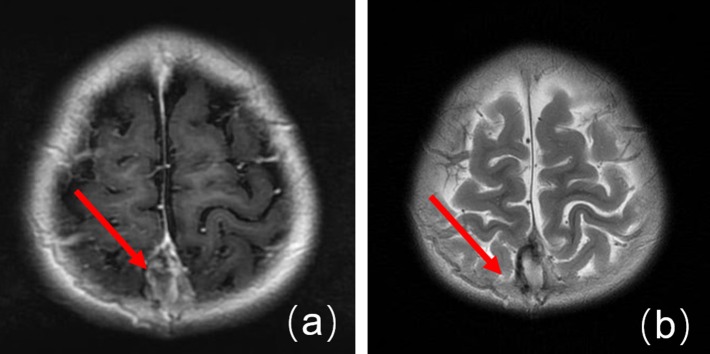
Enhanced MRI before treatment. **(a)** An MRI image showing a T1WI high-signal in the superior sagittal sinus, plus a slab-like low signal. **(b)** An MRI image showing a T2WI high-signal in the superior sagittal sinus.

The information system in our hospital was updated in 2012 and some data were missing. Therefore, the imaging and laboratory data were only obtained for 20 patients. Some patients initially received CT and MRI and were further confirmed by MRV. We found that (1) for the head CT examination, the density of the anterior sagittal sinus was raised in 2 cases (Figure 1, patient 30), and CVST diagnoses were confirmed by head MRV (Figure 2, patient 30). (2) Five cases for which head MRI examination was performed were diagnosed as CVST (Figure 3, patient 30). (3) Thirty patients with brain MRV were diagnosed with CVST (17 of them combined with enhanced scan). (4) Of the 20 cases with MRV data, 15 cases of superior sagittal sinus, 2 cases of inferior sagittal sinus, 12 cases of left transverse sinus, 14 cases of right transverse sinus, 7 cases of left sigmoid sinus, 9 cases of right sigmoid sinus, 6 cases of straight sinus, and 1 case of cavernous sinus were observed. Parenchymal involvement was observed in 1 patient. The imaging results are summarized in Table 1.

### Laboratory Testing

All patients underwent lumbar puncture examination and showed increased cerebrospinal fluid pressure, but no obvious abnormalities in cerebrospinal fluid in routine, biochemical or pathogenic examinations. Hematological parameters and coagulation tests of the patients were generally within reference interval. One exception was D-dimer with a median of 1.65. D-dimer levels in all patients were higher than 0.30 mg/L, the upper limit of the reference interval.

### Treatment and Prognosis

All patients were treated with low-molecular weight heparin anticoagulant therapy (90 U/kg each time, subcutaneously, twice daily). Twenty six patients had improved clinical symptoms, 2 patients died, and 2 were transferred to another hospital. Of the two patients that died, one deteriorated rapidly and eventually died due to intravenous sinusitis; the other had underlying diseases (lupus, moyamoya disease), which progressed rapidly after onset leading to death. Warfarin was used for 3 months after discharge; the initial dose was 0.1–0.2 mg/kg/d. Blood coagulation function was monitored every 2–4 weeks and warfarin dose was set to maintain the INR at 2.0–3.0. After a 3-month follow-up, 21 patients were found to have improved. No bleeding complications were observed. The clinical symptoms did not recur and visual acuity improved.

## Discussion

The clinical manifestations of CVST are complex and variable; they lack specificity, sometimes making it difficult to diagnose early. The literature reports that the early diagnosis and treatment of CVST could reduce the mortality rate to 5–15% (6). Therefore, the early diagnosis and treatment of CVST is currently the primary problem faced by neurologists. Although we evaluated only 30 cases in our study, we found that the morbidity of this disease is high in actual clinical practice and that only a few cases are diagnosed early. Only 10 of the 30 patients were diagnosed within 7 days of onset, suggesting that clinicians need to deepen their understanding of the disease so that an early diagnosis can be made and the rate of misdiagnosis could be reduced.

Pediatric CVST occurs due to both intravascular and vascular factors, and dehydration is a common risk factor for all ages. The literature reports that one-third to two-thirds of children with CVST are in a pre-thrombus state (7). In this study, 22 children with clinical manifestations of subacute or chronic headache exacerbations were found to be dehydrated when diagnosed with CVST, owing to the disease itself or the long-term use of dehydrating agents outside of the hospital. Seven infection-related cases presented with dehydration due to diarrhea. Eight children who developed nephritis or nephrotic syndrome (hormone therapy) were already in the pre-thrombus state and the occurrence of CVST was attributed to insufficient water intake due to vomiting. These findings are in accordance with those of several other studies in that infection, nephritis, or nephrotic syndrome may be important etiologies of CVST (8, 9). In this study, 63.3% (19/30) of the children presented with obvious cases of the disease, which is far lower than the 85% reported in international studies (10). These results suggest it is necessary to determine other causes, including anti-prothrombin deficiency and protein C or protein S deficiencies (11). The role of polycrystalline, coagulation, and fibrinolysis system gene polymorphisms in the pathogenesis of CVST is currently receiving increased attention.

Because of the different etiologies and locations of the affected sinus, the clinical manifestations of CVST are diverse and lack specificity. Thus, it is extremely difficult to diagnose CVST based on clinical manifestations alone. The disease could present with an onset that is acute, subacute, or chronic. An uncharacteristic headache is a common clinical symptom, and 90% of patients have complaints of a headache as reported from international papers (10). There were 25/28 children with headache in this study, 12/29 children with visual symptoms caused by increased intracranial pressure which led to decreased vision, and even blindness, followed by double vision caused by adductor nerve paralysis, and 9 cases of seizures, all of which were findings similar to those of international studies (10).

Neuroimaging is the principal basis for the diagnosis of CVST (12). Typically, an initial CT scan is performed; however, only a few cases present with characteristic features on CT scan such as the high-density shadow of the strip. After contrast enhancement, the delta sign (also called the empty triangle sign) features can be seen. Most children in this study presented with no obvious abnormalities with regard to the head CT examination. Only two cases that were found to have a high-density venous sinus were diagnosed as CVST. Therefore, it is not easy to rule out CVST from normal CT scan manifestations. Currently, CT venography (CTV) can be used to diagnose CVST if the patients have MRI contraindications (12). Head MRI and MRV are utilized to diagnose CVST with high sensitivity and specificity. During the subacute stage, T1WI and T2WI scans identified thrombus abnormalities essential for the diagnosis. MRV is characterized by a lack of cerebral venous sinus blood flow signal or marginal blurring, irregular lower signals, and venous collateral formation (13). Digital subtraction angiography (DSA) is considered to be the “gold standard” for the diagnosis of adult CVST. However, it is an invasive examination; thus, the compliance of pediatric case examination is limited (13). In this study, only three patients underwent a DSA examination, which found an intracranial venous sinus embolism. In recent years, with the development of MRI technology, scientists have gradually accepted the “gold standard” for CVST diagnosis which uses the combined application of enhanced MRI and MRV examination (14).

At present, the treatment for CVST is based primarily on adult cases. The purpose of CVST anticoagulant therapy is to avoid thrombus enlargement and contribute to spontaneous thrombolysis (15). Studies have shown that anticoagulant therapy can be safely applied to children, particularly when coagulation is monitored (16–18). The current consensus is that older children diagnosed with CVST without bleeding should receive anticoagulant therapy. It is safe and effective to administer subcutaneous injections of low-molecular weight heparin (2 times daily, 180 μ/kg/d) for 2 weeks, then switch to oral warfarin for 3 months to maintain an INR of 2.0–3.0. All patients in this study were administered low-molecular weight heparin anticoagulation therapy according to the INR; in 26 cases, clinical symptoms improved in a short period of time, whereas in 21 cases in which oral administration of warfarin was continued after discharge, no bleeding complications were reported, and there was no recurrence of clinical symptoms. Thus, anticoagulant therapy is considered safe and effective. For CVST in older children, knowledge from adult cases can be used to manage the symptoms of the acute phase and improve the prognosis based on systemic anticoagulant therapy combined with improved thrombolytic therapy and interventional therapy.

Because of the retrospective analysis of clinical data, there are some limitations in this study. The first limitation is that the definite underlying causes were not identified in 11 patients. Second, the follow-up time was only 3 months and a longer follow-up is needed in future studies. Third, genetic tests were not conducted in our institution; therefore, we were unable to obtain these data.

It is important to achieve an early diagnosis with CVST. Therefore, we believe that CVST should be highly suspected in the following clinical situations: (1) patients who have high risk factors for venous sinus thrombosis; (2) those with progressive intracranial hypertension, no obvious signs of neurolocalization, particularly those with normal CT scans; (3) sudden onset of focal system damage, such as due to cerebral infarction, but the lesion does not conform to the distribution of arteries, or the effect is not obvious after treatment according to the cerebral infarction; (4) there is no obvious cause of decreased vision, disturbance of consciousness accompanied by or without seizures, and the disease progresses rapidly; and (5) headache, vomiting, meningeal irritation, and cerebrospinal fluid changes that cannot be explained by other known causes such as encephalitis, meningitis, or bleeding.

In summary, CVST is prevalent in the pediatric population and etiology of this disease is complex. Because of the lack of specific clinical manifestations, the rate of misdiagnosis is high. Clinicians should carefully look for evidence of the disease, perform neuroimaging in a timely manner for early diagnosis, and give appropriate treatment as early as possible to improve prognosis and decrease complications.

## Data Availability

All datasets generated for this study are included in the manuscript/supplementary files.

## Author Contributions

X-HW contributed to the analysis, interpretation of the data, and drafting of the manuscript. L-MZ, Y-MC, JW, and L-FY performed the analysis and interpretation of the clinical data. S-ZZ conceived, designed, supervised the study, interpreted the data, and drafted and revised the manuscript content. All the authors read and approved the final manuscript.

### Conflict of Interest Statement

The authors declare that the research was conducted in the absence of any commercial or financial relationships that could be construed as a potential conflict of interest.
